# Spatiotemporal evolution of Chinese ageing from 1992 to 2015 based on an improved Bayesian space-time model

**DOI:** 10.1186/s12889-018-5417-6

**Published:** 2018-04-16

**Authors:** Xiulan Han, Junming Li, Nannan Wang

**Affiliations:** 1 School of Statistics, Shanxi University of Finance and Economics, Wucheng Road 696, Taiyuan, 030006 China; 2 Beijing Yihua Record Information Technology Co., Ltd. Hualu Senior Care and Health Management Co., Ltd, 138 Andingmenwai Street, Beijing, China

**Keywords:** Population ageing, Bayesian space-time model, Spatiotemporal evolution

## Abstract

**Background:**

Most countries are experiencing growth in the number and proportion of their ageing populations and this issue is posing challenges for economies and societies worldwide. The most populated country in the world, China, is experiencing a dramatic increase in its ageing population. As China is the world’s largest developing country, its serious ageing issue may have far-reaching effects not only domestically but also in other countries and even globally.

**Methods:**

In order to overcome the weaknesses of traditional statistical models and reveal further detail regarding the local area evolution, an improved Bayesian space-time model is presented in this paper and used to estimate the spatiotemporal evolution of Chinese ageing from 1992 to 2015.

**Results:**

The six eastern provinces with high levels of ageing have been experiencing an almost steady state, while Jiangsu, Shanghai and Zhejiang have weak increased trends of ageing, and the weak increased trend is decreasing. Although the northern and western provinces belong to the low ageing area, five of them have strong local growth trends and therefore strong potential to exacerbate ageing. Under the background of the “comprehensive two children” policy, the forecast value of China’s ageing rate is 13.80% (95% CI:11.24%,18.83% is) in 2030.

**Conclusions:**

Considering developments over the past 24 years, it has been determined that the areas of the Chinese mainland that are experiencing the highest levels of growth in ageing populations are the two central provinces, which are connected to seven eastern provinces and five southwestern provinces. High ageing areas are not only concentrated in the eastern provinces, but also include Sichuan and Chongqing in the southwest region and Hubei and Hunan of the central region. The seven provinces (municipalities or autonomous regions) of the central and western regions have both high ageing levels and strong growth rates, but the growth rate is decreasing.

## Background

The world’s population is continually ageing [1]. This increasing number of ageing people will pose major challenges in the future for health-care systems [2] and will have major implications for economies and societies, affecting labour markets, patterns of saving and consumption, social interactions, housing and transportation [3]. As the most populated country in the world, China’s total population reached 1.38 billion in 2016, accounting for nearly 19% of the global population [4]; the country has achieved the rank of an ageing society, with the overall population ageing rate reaching 10.8% in 2015 [5]. Because China is the world’s largest developing country, its serious ageing issue may have far-reaching effects not only domestically but also in social and economic development in other countries and even globally. The existing literature related to China’s ageing issue mainly focuses on two aspects: historical development and regional differences. Historical development studies on China’s ageing have revealed the trend of the nation’s deepening ageing. Based on population data from 1953 to 1994, Lai (1999) claimed that China’s ageing had begun following the comprehensive implementation of the family planning policy in 1974, and was followed by an accelerated trend [6]. Other researchers, in particular Zhang et al. [7], Banister et al. [8] and Zheng and Wei [9] found that China’s ageing level continues to increase and the population dividend period is disappearing, and that these trends will negatively impact the domestic and international economies. Studies examining regional differences in China’s ageing are related. Through analysis of the fourth, fifth and sixth census data, Wang et al. [10] found that significant regional difference existed in population ageing in China. Before 2000, the ageing population size and growth rate in the coastal areas were higher than those in the central and western regions. After 2000, with coastal areas overtaken by the central and western regions in terms of ageing growth rate, China’s population ageing begun spreading from east to west. Xiu-Li and Wang (2008) [11] found that the ageing of the eastern, central and western regions could be characterized as “high, medium and low”, respectively, with the differences between regions and provinces in the eastern region demonstrating a decreasing trend, although the overall inter-provincial differences were broadened. Based on the 2000–2010 yearbook data and spatial econometrics method, Ruyu et al. [12] argued that the regional spillover effect of population ageing and spatial heterogeneity in China were very significant, with the higher population ageing areas being mainly concentrated in the Yangtze River Delta and the Circum-Bohai Sea Region, with the overall national ageing growth rate having slowed. In addition, Zhou [13] studied the ageing problem in big cities based on the statistics of the fifth and sixth censuses in China, demonstrating the spatial distribution characteristics of the ageing populations of four mega cities in China (Beijing, Shanghai, Guangzhou, and Wuhan). Mai et al. [14] forecast China’s ageing rate and drew the conclusion that more than one-fifth to one-third of China’s population would be aged 65 and over by the end of 2050. Zhang et al. [15] shared this opinon and suggested that the population ageing rate would reach 32% in 2050.

The previous literature analyzed the ageing issue in China based mainly on census data, and employed the classical statistical model or the econometric model, which are both based on the principle of large sample inference and are too dependent on sample information, so the corresponding parameters are estimated to be biased if the sample is biased or sparse. However, Bayesian statistics make full use of the a-priori information, where the unknown parameters are regarded as random variables and the parameter estimation is given in the form of probability distribution. The Bayesian method takes the uncertainty of reality data into account and has a higher degree of confidence. It is particularly important to note that, for space-time data, there is only one observation at a certain time and space intersection, so it does not meet the large sample requirements of classical statistics. Moreover, due to Tobler’s First Law of Geography, there is a certain correlation between the samples in the space-time domain; that is, the space-time data has characteristics of a small sample and autocorrelation (non-independent), which pose serious challenges to the classical statistical model based on large sample inference. The Bayesian statistical model, however, overcame the problem of small sample size and estimates parameters in the form of probability distributions under consideration of various uncertainties, by introducing the a-priori information and taking full advantage of the autocorrelation characteristics of data in the spatiotemporal domain.

This paper aims to investigate the situation in China with regard to the evolution of spatio-temporal characteristics of the ageing population, the spatial heterogeneity of the ageing level of the population, and the local trends in population ageing. Consequently, the present study forecasts the value of China’s ageing rate against the background of the “comprehensive two children” policy. These results can provide a reference for relevant policy makers, public health managers and demographers. In order to overcome the weakness of traditional statistical models and reveal further detail on local area evolution, the Bayesian space-time model is extended and estimated on the basis of Chinese provincial statistics data (1992–2015). Based on this, China’s ageing rate in 2030 is also predicted under the background of the comprehensive “two children” policy.

## Methods

### Source data

The data used in this paper are provincial population data drawn from the China Demographic and Employment Statistics Yearbook 1993–2016, including the 2000 and 2010 data sets, which are derived from the fifth and sixth national census, covering 31 provincial-level administrative regions in mainland China and excluding the regions of Hong Kong, Macao and Taiwan. The rate of ageing, the key focus of this paper, refers to the proportion of the resident population aged 65 and above compared to the total resident population in a certain region.

### Bayesian space-time hierarchical model

The Bayesian space-time hierarchical model (BSTHM) is a combination of the Bayesian hierarchical model and the spatio-temporal interaction model [16, 17]. The general form is:

Data Model: y_it_~*P*(*y*_*it*_(*θ*_*it*_, Θ)| Θ) (1).

Process Model: θ_it_ = *S*_*i*_ + Λ_*t*_ + Ω_*it*_ + *ε*_*it*_ (2).

Hyperparameters Model: Θ~P(Θ) (3).

where y_it_ denotes the observational value, θ_it_ is the space-time dependent variable, and *S*_*i*_ and Λ_*t*_ represent the common spatial state and overall time trend, respectively, Ω_it_ measures the time and space interaction effect, *ε*_*it*_ is random noise and is the hyperparameters set.

Considering that the ageing population observational data is count data and possibly over-dispersed, the data model this paper adopts is the Poisson-Gamma hybrid model [18]:

4 $$ {\mathrm{y}}_{\mathrm{it}}^{\mathrm{ageing}}\sim Possion\left({n}_{it}{p}_{it}^{aging}{u}_{it}\right) $$

5 $$ {\mathrm{u}}_{\mathrm{it}}\sim Gamma\left({r}_{it},{r}_{it}\right) $$

Where ${\mathrm{y}}_{\mathrm{it}}^{\mathrm{ageing}}$ is the population aged 65 and above, *n*_*it*_and ${p}_{it}^{aging}$are the total population and the rate of ageing in region i(i = 1, 2, …, 31) in year t, respectively, u_it_ are random effect parameters and *r*_*it*_ is the divergence coefficient [18]. In the existing BSTHM [16], the process model is:

6 $$ \ln \left({p}_{it}^{aging}\right)=\upalpha +{\mathrm{s}}_{\mathrm{i}}+\left({b}_0\mathrm{t}+{v}_t\right)+{b}_{1i}t+{\varepsilon}_{it} $$

Where α is the basic fixed constant for national overall ageing, s_i_ is the common spatial risk of ageing in the overall province trend, and (*b*_0_t + *v*_*t*_) consists of a linear trend *b*_0_t and a random effect, *v*_*t*_, allowing for nonlinear variation. *b*_1*i*_ denotes the local tendency in local regions, which is isolated from the overall trend. *ε*_*it*_ is a Gaussian random variable.

### Improved Bayesian space-time model

In the existing BSTHM, the non-linear overall trend is considered but the non-linear local trend is neglected. In view of this, this paper improved the BSTHM by adding a quadratic term to the process model, which describes the second-order change of the local trend, that is, acceleration. Thus, more detailed information pertaining to the time and space interaction effect can be abstracted and the elaboration of local trends can be realized. The above formula (6) is revised as:

7 $$ \ln \left({p}_{it}^{aging}\right)=\upalpha +{\mathrm{s}}_{\mathrm{i}}+\left({b}_0\mathrm{t}+{v}_t\right)+\left({b}_{1i}t+\frac{{\mathrm{b}}_{2\mathrm{i}}{\mathrm{t}}^2}{2}\right)+{\varepsilon}_{it} $$

Where b_2i_ is the second-order variation factor of the local tendency in the space-time process, and its physical meaning, acceleration, will be further elaborated with the statistical results. The meanings of the other parameters are the same as in eq. (6).

This paper introduces the Besag York Mollie (BYM) model [19] to determine the prior distribution of s_i_, b_1i_ and *b*_2*i*_, using a conditional autoregressive (CAR) normal prior form to express the spatial structured and unstructured random effects. The spatial adjacency matrix adopts the first-order “queen” adjoining form. The prior distribution of time random effects parameters also uses CAR normal prior form, in which time adjacency adopts a one-dimensional first-order adjacency matrix. According to the conclusion of Gelman (2006) [20], the prior distribution of mean square error for all random variables in the model is determined as a strictly positive half-Gaussian distributionN_+∞_(0, 10).

In this paper, Bayesian statistical estimation is achieved by WinBUGS [21] based on the MCMC method. Two MCMC chains are used to ensure the convergence of the model and the number of iterations for each chain is set to 250,000, of which 200,000 are for the burn-in period and 50,000 are for the number of iterations of posterior distribution for parameter estimation. The convergence is evaluated with the Gelman-Rubin statistical parameter; the closer the value is to 1, the better the convergence is [22]. The Gelman-Rubin parameters of all the parameters in this study range from 0.9988 to 1.0013, indicating that the convergence of this statistical result is good.

### Ageing rate prediction method

We study the spatio-temporal evolution of China’s total population ageing based on the population data of 1992–2015. The improved Bayesian space-time model is:

8 $$ {\mathrm{Y}}_t^{\mathrm{ageing}}\sim Possion\left({n}_t{P}_t^{ageing}{U}_t\right) $$

9 $$ {\mathrm{U}}_{\mathrm{t}}\sim Gamma\left({r}_t,{r}_t\right) $$

10 $$ \ln \left({\mathrm{P}}_{\mathrm{t}}^{\mathrm{ageing}}\right)=\upalpha +\left({\mathrm{b}}_0\mathrm{t}+{\mathrm{V}}_{\mathrm{t}}\right)+\left({\mathrm{b}}_1\mathrm{t}+\frac{{\mathrm{b}}_2}{2}{\mathrm{t}}^2\right)+{\upvarepsilon}_{\mathrm{t}} $$

Where ${\mathrm{Y}}_t^{\mathrm{ageing}}$, *n*_*t*_ and ${P}_t^{ageing}$denote the ageing population, total population and ageing rate in year t, respectively, with the meaning of the other parameters being the same as in eq. (7). The spatial statistical unit corresponding to this prediction model is overall and therefore without lower right corner mark i.

After estimating the parameters of the above model based on the 1992–2015 data, China’s ageing rate in 2030 can be predicted. Considering that the Chinese government began implementing the comprehensive “two children” policy in 2016, this will result in an additional increase in the total population, although it will not alter the size of China’s ageing population over the next 30–40 years, thereby slowing the trend of ageing [17]. Therefore, in the context of policy implementation, the above-predicted rate of ageing needs to be corrected. Assuming thatP_2030all_ is the predicted total population by the end of 2030 in China without implementation of the new population planning policy and that P_2030up_is the predicted increase in the total population by the end of 2030 under the conditions of new policy implementation, in the context of the new comprehensive “two children” policy, the predicted value calculation formula for the ageing rate by the end of 2030 in the Chinese mainland is:

11 $$ {\mathrm{R}}_{2030}^{\mathrm{ageing}}=\frac{{\mathrm{P}}_{2030\mathrm{all}}^{\mathrm{ageing}}{\mathrm{P}}_{2030}^{\mathrm{ageing}}}{{\mathrm{P}}_{2030\mathrm{all}}^{\mathrm{ageing}}+{\mathrm{P}}_{2030\mathrm{up}}^{\mathrm{ageing}}} $$

## Result

### Descriptive statistical result

**Fig. 1:**
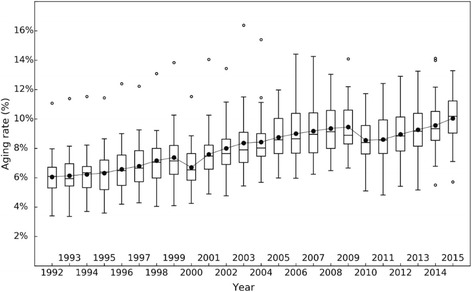
Boxplot of the ageing rate in the Chinese mainland from 1992 to 2015

During the 1992–2015 period, the overall ageing rate in mainland China maintained a sustained rise. The percentage of the population aged 65 and above was 6.08% in 1992 and grew to 10.47% in 2015, with an average annual growth rate of 2.29%. The extent of ageing has been increasing year by year (Fig. 1). The entire process can be divided into three sub-processes: 1992–1999, 2000–2010 and 2010–2015. The increasing tendency of the mean of three stages increased stage by stage, and the high quartile and low quartile also had some degree of increase.

**Fig. 2:**
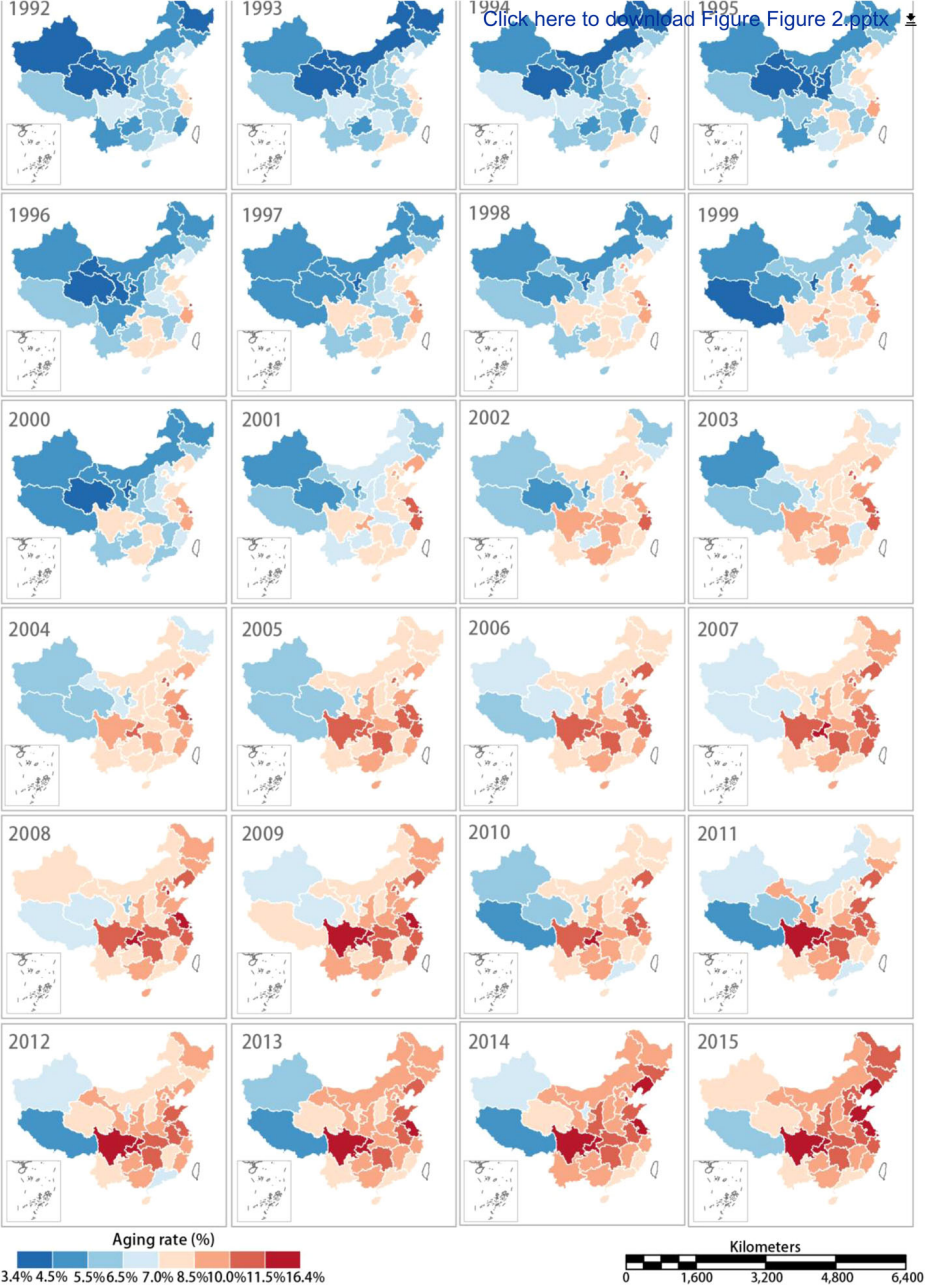
Spatial distribution of the ageing rate in the Chinese mainland from 1992 to 2015. (Map generated with ArcGIS 10.3 by authors)

At the same time, the ageing area showed a significant expansion trend. According to the lower limit of the Ageing Social Standard of the United Nation (7.0%) [23], the following five provinces (municipalities) became ageing areas as early as 1992:Shanghai (11.%), Beijing (8.0%), Zhejiang (7.5%), Tianjin (7.4%) and Jiangsu (7.4%). Thirty provinces, excepting Tibet, became ageing areas in 2015. Of these, Chongqing had the highest rate of ageing (13.3%). Figure 2 shows the evolution process of the spatial distribution of the ageing rate of 31 provinces (including some municipalities or autonomous regions; for brevity, the following text only uses the 31 provinces) in China during the study period, illustrating a clear expansion from southeast to northwest, which is consistent with the conclusions of Wang et al. [10]

**Fig. 3:**
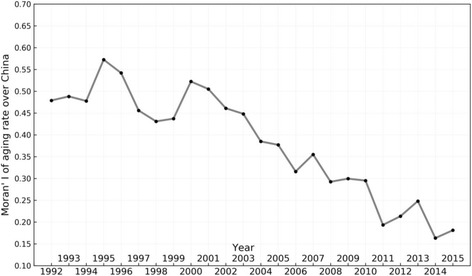
Moran’s I of the ageing rate in mainland China from 1992 to 2015

We use Moran’s I [24] to measure the spatial difference of the ageing rate; the larger the Moran’s I is, the larger the spatial difference is. Figure 3 shows the trend of the spatial variation coefficient of the ageing rate in 31 provinces in mainland China from 1992 to 2015. It can be observed that the differences in ageing demonstrate a general decreasing trend. The difference in degree of ageing among provinces was quite large during 1992–2004, and decreased during 2004–2015, which is contrary to the conclusions of Xiu-Li and Wang. [8] In recent years, as can be seen in Fig. 1, the degree of ageing in each province has been aggravating. All regions attained a high ageing rate, so a smaller spatial difference resulted.

### Bayesian statistical result

In order to explain the spatio-temporal evolution of the ageing rate in mainland China more thoroughly, this paper divides the research period into three stages (1992–1999, 2000–2010 and 2010–2015) based on the above descriptive statistical analysis, and then, using the extended nonlinearity BSTHM, estimates the common spatial pattern, the distribution of local first-order change (speed) and second-order change (acceleration) in the three sub-phases of the ageing rate in China.

### The common spatial pattern

**Fig. 4:**
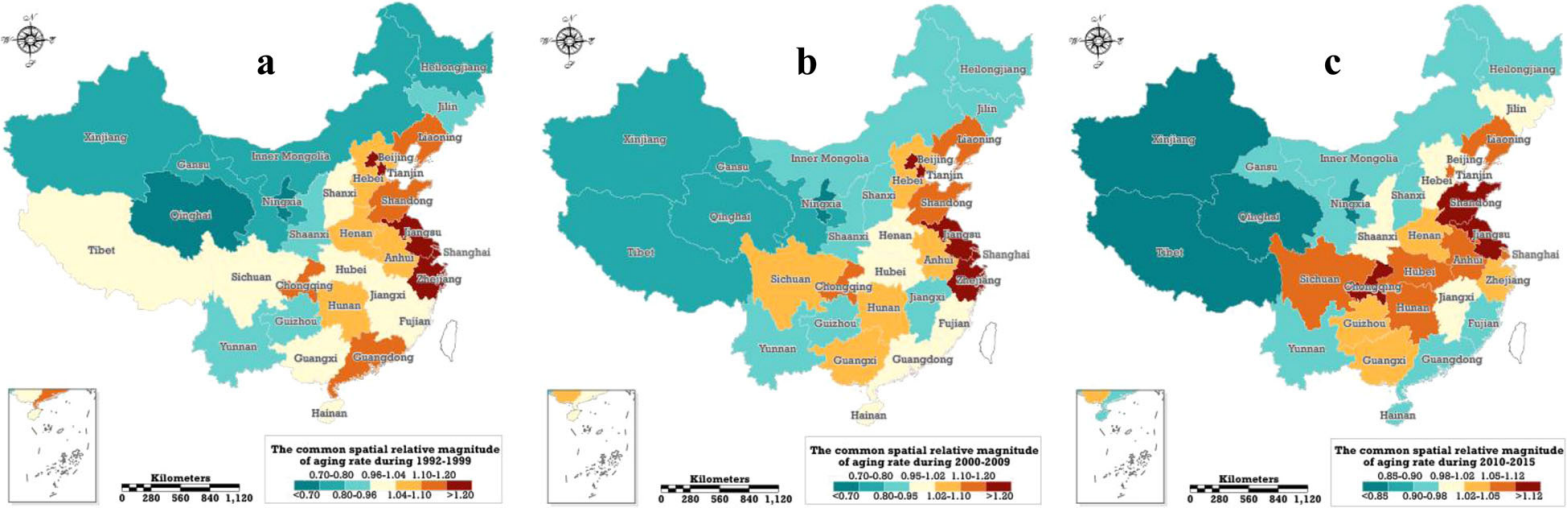
The common spatial relative magnitude of the ageing rate from 1992 to 1999 (**a**), 2000–2009 (**b**) and 2010–2015 (**c**), the posterior medians of the parameter exp(s_i_), the values measure the corresponding relative magnitude of the provinces’ ageing levels relative to the overall national level, exp(α), in the sub-period. (Map generated with ArcGIS 10.3 by authors)

Based on this paper’s improved nonlinearity BSTHM, the posterior median estimation of the steady-state spatial relative magnitude of 31 Chinese provinces, exp(S_i_), in three sub-periods can be obtained; this value measures the relative magnitude of the provinces’ ageing levels relative to the overall national level, exp(α). If exp(S_i_) > 1.0, it indicates that the degree of population ageing in a province is exp(S_i_) times the overall level, and vice versa. Figure 4 shows the common spatial pattern in the three periods of 1992–1999, 2000–2010 and 2010–2015.

Overall, in the eastern provinces, especially in the six regions of mainland China of Beijing, Tianjin, Shandong, Jiangsu, Shanghai and Zhejiang, the degree of ageing is much higher than the overall level during the three periods. In the northwestern provinces, especially Xinjiang, Qinghai, Gansu, Inner Mongolia, Heilongjiang and Ningxia, the degree of ageing is lower than national level during the three periods. This is basically consistent with the conclusions of Wang et al. [10]、Xiu-Li and Wang [11]. and Ruyu et al. [12]

Specifically, certain provinces have unique characteristics during the three stages. Guangdong province, as the most populous province in mainland China (1.04 billion, 2010 population census), had its ageing level show a sequential decrease in three stages: during 1992–1999, the ageing degree was 1.139 times the national overall level, while during 2000–2009 and 2010–2015, the ageing level was at and below the national overall level, respectively, and the spatial relative magnitude of ageing was reduced to 1.012 and 0.920, respectively. Shandong province, as the second-most populous province (95.79 million, 2010 census) maintained a relatively high level of ageing over the three phases, with a spatial relative magnitude of 1.147, 1.143 and 1.129, respectively. Although Sichuan and Chongqing are located in the underdeveloped southwestern region, their population ageing problems were more serious than in the other southwestern provinces, which may be related to population movements. According to previous related studies, in these two regions a large number of young and middle-aged laborers have left in recent years [25], leading to a continuous ageing of the population. However, the ageing in Sichuan and Chongqing had different staged characteristics; the ageing in Sichuan was still on an average level (with a spatial relative degree of 1.015) during 1992–1999, and significantly higher than that of mainland China after 2000. Chongqing’s ageing level was higher than that in Sichuan in all three stages, as its ageing spatial relative magnitude was consistently above 1.10 and reached a peak value of 1.187 in 2010–2015. It should be pointed out that, in the three municipalities of Beijing, Tianjin and Shanghai, the ageing of the population was at a high level during the first two sub-periods, but overtaken by Chongqing, Jiangsu and Shandong during the last sub-period. In addition, the level of ageing in Jiangsu Province was higher than the average over the entire study period, while in Zhejiang province it was somewhat lower during the third stage. Ageing in the western and northwestern regions and Yunnan province was always lower than the average during the entire study period. An interesting phenomenon is that following the most recent 24 years of evolution, the high-level ageing areas of mainland China have spatially located in the two central provinces, connecting to seven eastern provinces and five southwestern provinces, as shown in Fig. 4. Although Wang et al. [10], Xiu-Li and Wang [11]. and Ruyu et al. [12] also pointed out that differences existed in inter-provincial ageing, they did not systematically and thoroughly study the relative ageing levels of provinces in various stages. This paper argues that, in the context of ageing rising across all regions, it is more scientific to study the matter from a relative perspective.

### Local evolution trend

Based on the improved BSTHM, the paper achieves a more detailed estimation of the local evolution trend, including the first and second order trend. The former, denoted by b_1i_ in equation (10), is equivalent to the growth rate in physics, whereas the latter, denoted by b_2i_ in equation (10), is equivalent to acceleration and measuring the change of the former. b_1i_ > 0 (b_1i_ < 0) indicates that the province *i* belongs to the area with strong (weak) ageing growth rate, with the ageing growth rate being stronger (weaker) than the national general growth rate. Different combinations of b_1i_ and b_2i_ mean different local evolutionary characteristics: b_1i_ > 0 and b_2i_ > 0 means that province *i*’s ageing has a strong increase trend and the strong increase will become stronger; b_1i_ > 0 and b_2i_ < 0 indicates that province *i*’s ageing has a strong increase trend but the strong increase will become not strong; b_1i_ < 0 and b_2i_ > 0 means that the ageing in province *i* was weak, but the weak increase will transform into strong growth; b_1i_ < 0 and b_2i_ < 0 mean that the ageing in province *i* has a weak growth trend and the weak growth will become weaker.

**Fig. 5:**
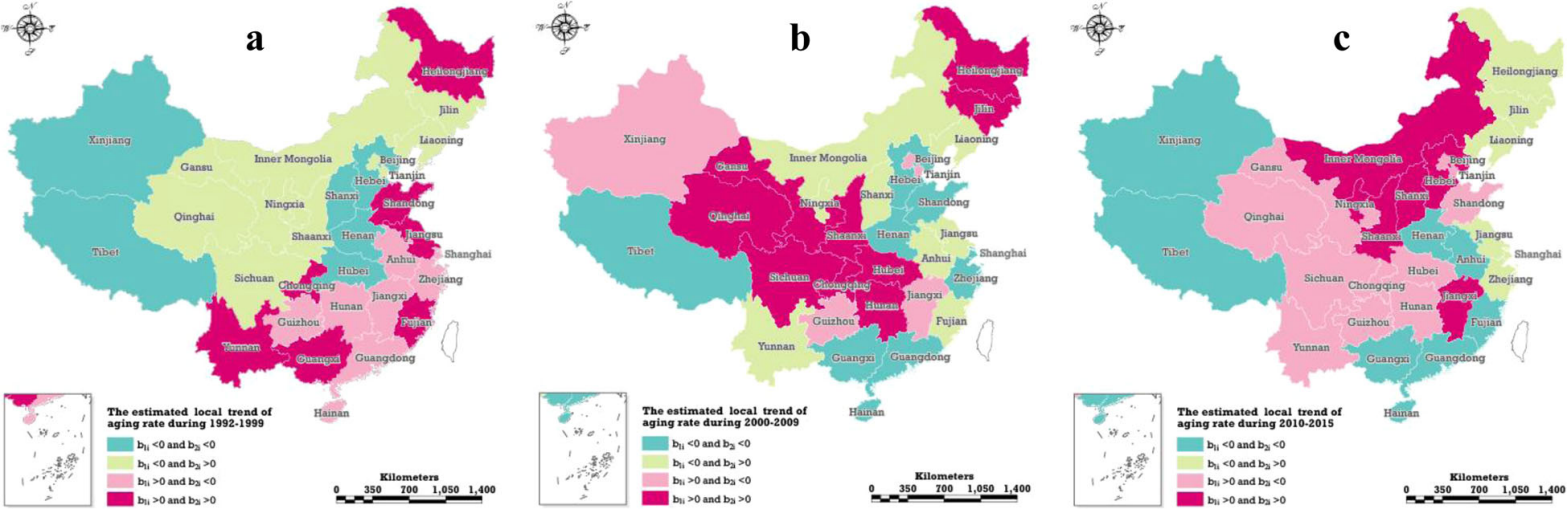
Local ageing rate trends from1992–1999 (**a**), 2000–2009 (**b**) and 2010–2015 (**c**), the posterior medians of the parameters, b_1i_, b_2i_, estimated using the developed Bayesian space-time model. (Map generated with ArcGIS 10.3 by authors)

Based on the comprehensive consideration of the common spatial effect, overall time effect and the time-space interaction effect, this paper estimates the local evolution trend of ageing in 31 Chinese provinces in three stages, and for the first time estimates the change in aging growth within each province, thereby providing a detailed description of the spatial and temporal evolution of ageing in mainland China. Figure 5 is a graphical representation of the local evolution characteristics of ageing in 31 provinces in mainland China in the three phases of 1992–1999, 2000–2009 and 2010–2015.

From 1992 to 1999, the areas of vigorous increasing ageing in mainland China were mainly distributed in the eastern and southern regions. Among them, six provinces (the middle-eastern coastal areas including Shandong, Jiangsu and Fujian and the southwestern provinces including Chongqing, Yunnan and Guangxi) had ageing that showed a tendency to surpass the overall growth rate, while the growth rate of ageing in 7 provinces (Guizhou, Hunan, Guangdong, Jiangxi, Anhui, Zhejiang and Shanghai) was higher than the overall average, although the growth rate was diminishing and approaching the overall trend. Both central and western provinces belong to the weak growth areas, especially in central China, including Hebei, Shanxi, Henan and Hubei, but also in western regions such as Xinjiang and Tibet. In these regions, not only was the ageing growth rate below the overall average, but also the gap was widening.

From 2000 to 2009, the areas of strongest increased ageing shifted from the eastern and southern regions to the central and western regions, with 14 strong growth provinces emerging. Among them, nine provinces, including Sichuan, Hubei and Henan, among others, experienced accelerated ageing growth rates, In five provinces (Beijing, Tianjin, Jiangxi, Guizhou and Xinjiang) the ageing growth rate demonstrated a slowing trend. The strong growth of ageing in the eastern region expanded from Heilongjiang to Jilin, with both located within to the accelerated growth area. The eastern and southern provinces, with the exceptions of Beijing and Tianjin, were all transformed from strong growth of ageing areas to weak growth of ageing areas. With the, exceptions of Jiangsu and Fujian, all five provinces experienced acceleration toward the overall trend in ageing growth rate.

Compared with the previous two phases, from 2010 to 2015 the number of ageing strong growth provinces increased significantly, reaching 16, which exceeded 50% of the total number of provinces in mainland China. With the exception of Hebei, Shandong, Beijing and Tianjin, which are located in the eastern region, most of the strong growth provinces are located in the central and western regions. Simultaneously, the provinces with accelerated ageing growth rates were Hebei, Inner Mongolia, Shanxi, Gansu, Ningxia and Jiangxi, numbering six in total, fewer than the second stage. According to the common spatial pattern of ageing as shown in Fig. 4, the eastern provinces, with high degrees of ageing, are among the weak ageing growth provinces.

### Prediction of ageing in mainland China in 2030

Before prediction, the reliability of the model must be tested. In this paper, cross-validation is used for this purpose. Specifically, the data on the ageing rates of the three five-year periods (1995–1999, 2002–2006 and 2010–2014) are extracted separately and used as test truth data only, not in the calculation process. The remaining 19 years of data are used to estimate the rate of ageing of the test year. Then, we calculate the root mean square error (RMSE) between the estimated and observed values. In this paper, the RMSE of the prediction of the ageing rate during three periods is 0.71%, 0.53% and 0.62%, respectively, all less than 1%, so the model prediction error is within a reasonable range.

Under the premise of maintaining the “one child” policy unchanged, based on 1992–2015 population data, the population ageing rate in 2030 ${p}_{2030}^{aging}$

is projected to be 14.76% (95%CI: 12.02%, 20.13%). In the context of the comprehensive “two-child” policy, the total population in 2030 is projected to be 1.45 billion by the National Population Development Plan (2016–2030). According to Zhai et al. [26], the estimated value of the total population increase is 94 million. Therefore, without implementation of the comprehensive “two-child” policy, the predicted total population in 2030 is 1.36 billion. According to the above formula (10), considering the context of the comprehensive “two-child” policy, the prediction value of the ageing rate in 2030 in mainland China is 13.80% (95% CI: 11.24%, 18.83% is). This result means that the rate of ageing in China decreased by 0.96% (95% CI: 0.78%, 1.30%) in 2030 after the implementation of the comprehensive “two-child” policy.

## Discussion

This paper focuses on exploring the temporal and spatial evolutionary characteristics of ageing in mainland China. In order to overcome the weaknesses of traditional statistical models and reveal further detail of the local area evolution, the Bayesian space-time model is extended and estimated on the basis of Chinese provincial data (1992–2015). According to the box plots, the entire study period is divided into three stages: 1992–1999, 2000–2009 and 2010–2015. In this paper, an improved BSTHM was used, considering the nonlinear characteristics of local trends and decomposing them into two parts. The first-order (growth rate) and second-order changes (acceleration, measuring the change of the local growth rate) are used to estimate the common spatial pattern and local trend of China’s ageing population in three stages.

According to the results of this paper, population ageing has become a common problem in 31 provinces in mainland China. During the period from 1992 to 2015, the level of ageing in each province showed a continuous upward trend, but the change in trend was significantly varied, so the differences in the ageing levels among different provinces show a trend of decreasing gradually over the recent 24-year period. At present, the spatial distribution of high-ageing areas consists of 14 provinces, including Shandong, Jiangsu, Shanghai, Anhui, Hubei, Chongqing, Sichuan and Hunan, among others. These provinces are all economically developed (or relatively so) and have large population areas. According to the 2010 census data, the ageing population in these 14 provinces accounted for 63.94% of the ageing population in mainland China. This indicates that both the number of older people and the old-age dependency ratio have been increasing significantly. At the same time, the labour force population, that is, the labour supply, has been decreasing, meaning that the demographic dividend, which is an important factor in maintaining rapid economic development, is fading out. The increasing numbers of older people may result in higher social medical costs, particularly for drugs and the treatment of chronic illnesses. This will lead to a transfer in the social medical source configuration. According to the results of this paper, in addition to the 14 provinces, other provinces, even some in the west, will also face serious ageing issues as these relative low ageing regions have even higher trends in population ageing.

It should be pointed out that Guangdong Province, as the largest province in terms of population, has seen its ageing rate continue to increase. However, its ageing rank order in mainland China has been declining continuously and was lower than the overall ageing of the population from 2010 to 2015. This is in contrast to the characteristics of other eastern coastal provinces and should be attributed to large population inflows. Since the inflow population mostly consists of young and middle-aged members of the labor force, the level of ageing of the provinces with net population inflow is thereby reduced. According to a study conducted by Qiao et al. [25], the migrant population flowing into Guangdong accounts for 25.03% of the total floating population in China. The influx of a large number of young people makes the level of ageing in Guangdong lower than the overall national level. In addition, Fujian province, located in the eastern coast of China, has a rate of ageing at a low level within mainland China. This can also be attributed to the inflow of population. Fujian province ranks 17th in total population, but its migrant inflow is sixth in mainland China (2010 Census) [25]. The large inflow of non-ageing population largely dilutes the level of ageing and explains why the level of ageing in Fujian province is also lower than the overall level within mainland China.

Although this paper extends the BSTHM and reveals further details regarding the temporal and spatial variation of ageing in mainland China over a recent 24-year period, it still has certain defects. First, the population data were generated two different ways. The 2000 and 2010 datasets, the 5th and 6th national demographic censuses, were collected using a full survey. Datasets for the other years were produced by proportional sampling with multi-hierarchy, multistage and whole group probability. The accuracy of the census data is higher than that of the sampled data. Still, the Bayesian statistical method, BSTHM, which considers greater more uncertainties can to some extent overcome the limitation of the diverse accuracies of the input data. Second, this paper takes the provincial area as the spatial statistical unit, which is not fine enough in terms of spatial granularity. If city or even county data were available and if the spatial statistical unit were to be further subdivided, the problem could be studied in greater detail. Third, this article only discusses the temporal and spatial evolution of the ageing problem itself, but does not study the formation mechanisms and influencing factors. This is the direction in which we will direct future studies.

## Conclusions

The main findings of this study include the following: (i) After a recent 24-year period of development, the Chinese mainland’s high ageing areas have distributed in the central two provinces, connecting to seven eastern provinces and five southwestern provinces. High ageing areas are not only concentrated in the eastern provinces, but also include provinces in southwestern Sichuan and Chongqing as well as central Hubei and Hunan. Chongqing and Sichuan have especially high rates of ageing, with their ageing rates in 2015 being 13.3% and 12.9%, respectively, making them the highest and second highest, respectively, in China; (ii) The high rates of ageing in the eastern provinces have been in an almost steady state, with slightly increased levels and decreased growth rates in the ageing rates in Jiangsu, Shanghai and Zhejiang. The ageing rate in Guangdong and Fujian (two eastern coastal provinces) has been reduced to below that of the overall level of the Chinese mainland; (iii) High ageing areas are not only concentrated in the eastern provinces, but also include Sichuan and Chongqing in the southwest region and Hubei and Hunan of the central region; (iv) The seven provinces (municipalities or autonomous regions) of the central and western regions belong to both the high ageing levels and strong growth rate areas, but the growth rate is decreasing; (v) The northern and western provinces belong to the low ageing area, but five of them have strong local growth trends and so have great potential for ageing to be exacerbated; (vi) With the background of the comprehensive “two children” policy, the forecast value of China’s ageing rate is 13.80% (95% CI:11.24%,18.83% is) in 2030.

Most countries in the world are facing similar ageing issues. The formulation of strategic policies and the allocation of public resources to deal with ageing problems are urgently required to recognize more detailed characteristics of ageing in time and space. We hope that this paper’s research on the spatial differences and trends of local changes in ageing will provide a meaningful reference for other countries attempting to deal with the ageing problem. Based on the results of this study, it is suggested that when formulating strategic policy to deal with ageing, we should take full account of the regional differences and trends of local changes of various regions. Different policies should be formulated based on local conditions in order to scientifically deal with the social and economic problems brought about by ageing.

## Abbreviations

BSTHM: Bayesian space-time hierarchical model
CAR: Conditional auto-regression
CI: Confidence interval
MCMC: Markov Chain Monte Carlo
